# Understanding the Economic Value of Molecular Diagnostic Tests: Case Studies and Lessons Learned

**DOI:** 10.3390/jpm3040288

**Published:** 2013-10-25

**Authors:** Adrian Towse, Diego Ossa, David Veenstra, Josh Carlson, Louis Garrison

**Affiliations:** 1 Office of Health Economics, London SW1E 6QT, UK; 2 Novartis Pharma AG, Novartis Campus, Basel CH-4002, Switzerland; E-Mail: diego.ossa@novartis.com; 3 Pharmaceutical Outcomes Research & Policy Program, School of Pharmacy, Department of Pharmacy, University of Washington, Seattle, WA 98195, USA; E-Mails: veenstra@uw.edu (D.V.); carlsojj@uw.edu (J.C.); lgarrisn@uw.edu (L.G.)

**Keywords:** diagnostics, companion-diagnostics, personalized medicine, clinical utility, economic value, decision-making

## Abstract

Ten years after completion of the Human Genome Project, progress towards making “personalized medicine” a reality has been slower than expected. The reason is twofold. Firstly, the science is more difficult than expected. Secondly, limited progress has been made in aligning economic incentives to invest in diagnostics. This paper develops nine case studies of “success” where diagnostic tests are bringing personalized medicine into clinical practice with health and economic impact for patients, healthcare systems, and manufacturers. We focus on the availability of evidence for clinical utility, which is important not only for clinicians but also for payers and budget holders. We find that demonstrating diagnostic clinical utility and the development of economic evidence is currently feasible (i) through drug-diagnostic co-development, and (ii) when the research is sponsored by payers and public bodies. It is less clear whether the diagnostic industry can routinely undertake the work necessary to provide evidence as to the clinical utility and economic value of its products. It would be good public policy to increase the economic incentives to produce evidence of clinical utility: otherwise, opportunities to generate value from personalized medicine—in terms of both cost savings and health gains—may be lost.

## 1. Introduction

Ten years after completion of the Human Genome Project, progress towards making “personalized medicine” a reality has been slower than expected [1]. The reason is twofold [2]. Firstly, the science is more difficult than we expected, for example, the limitation of genetic prediction *vis-à-vis* a patient’s response to a drug. Secondly, little progress has been made in aligning economic incentives to invest in diagnostics. Existing regulatory and reimbursement practices have not created an environment that sufficiently rewards diagnostic manufacturers for generating the evidence of clinical utility and cost-effectiveness that payers are often looking for. The result is often a paucity of direct or relevant evidence.

Despite these challenges, the knowledge emerging from the Human Genome Project and its application through molecular diagnostic (MDx) technologies are producing some benefits for patients and health systems. However, understanding the conditions that favour the development of evidence is challenging. The objective of this paper was to identify how evidence has been generated by critically evaluating successful case studies, and, to the extent possible, identify any lessons from the case studies.

Through nine case studies we identified examples of “success” where diagnostic tests are bringing personalized medicine into clinical practice with positive health and economic impact for patients, healthcare systems, and manufacturers. We judged success according to the ability to deliver one or more of: information of value; targeting of treatment; improvement in health status; cost offset; and the avoidance of adverse reactions. These cases illustrate the diversity of MDx technology, and highlight both the potential for value and the key difficulties that have emerged. In particular, we focus on the nature of any associated evidence of clinical utility that might facilitate the decision-making process not only for clinicians but also for payers and budget holders. We believe the findings of this paper will be helpful for policy makers and MDx developers in ascertaining how the circumstances in which good evidence of clinical utility can be generated. 

## 2. Nine Case Studies of MDx in Personalized Medicine

Based on a review of the literature and our knowledge of trends in the field we chose nine case studies to show the diversity of MDx, its potential value in personalized medicine, and the key difficulties that have emerged. There are a limited number of examples in the literature. Using our knowledge of the field we sought to focus on a manageable number of case studies chosen to reflect as much diversity as was feasible. They represent prominent examples of MDx covering a spectrum of clinical applications in the use of MDx and pharmacogenomics (PGx), ranging from targeting cancer treatment to diabetes risk testing. 

The majority of the case studies are in oncology, which is the area with the most development activity and clinically available applications to date. The prominence of cancer diagnostics reflects the importance of genomic variation in the genesis of cancer and the role that specific variations play as therapeutic targets. The five are: (1) Oncotype Dx^®^ and MammaPrint^®^ gene expression testing for breast cancer recurrence; (2) human epidermal growth factor receptor type 2 (HER2) in breast cancer (BrCa); (3) EGFR mutation testing in non-small cell lung cancer (NSCLC); (4) KRAS mutation testing in colorectal cancer (CRC); and (5) BCR-ABL monitoring testing in chronic myeloid leukaemia (CML). The remaining four cases are: testing for the CYP2C19 enzyme which reduces the effectiveness of the oral antiplatelet agent clopidogrel (Plavix^®^); testing for the HLA-B*5701 allele for HIV treatment with abacavir; testing for viral load monitoring (VLM) to manage the treatment of hepatitis C; use of the PreDx^®^ Diabetes Risk Score (DRS) in Type-2 Diabetes.

We first describe the clinical use and evidence supporting each of the nine case studies, and then summarize the variations among them in terms of the evidence base.

### 2.1. Oncotype DX^®^ and MammaPrint^®^ Testing in Early Stage Breast Cancer

Breast cancer (BrCa) is the most commonly diagnosed cancer in women. Traditionally, clinical, histological and molecular factors such as oestrogen receptor (ER) expression and HER2 overexpression are considered when assessing risk and recommending therapies [3]. Through the assessment of prognostic and predictive factors, gene expression profiling can also assist in the “personalisation of BrCa treatment” by improving the identification of patients who will gain most benefit from the therapy [4].

Oncotype DX^®^ and MammaPrint^®^ are gene expression profiles used for prognosis and/or prediction in early stage BrCa treatment. Oncotype DX^®^ measures the expression of 21 genes and generates an individualized “recurrence score” that predicts the risk of recurrence in women with lymph node negative, ER-positive early stage BrCa, and identifies those most likely to benefit from adjuvant chemotherapy. The assay development was based on clinical samples from the National Surgical Adjuvant Breast and Bowel Project (NSABP) B-20 clinical trial [4], and subsequently validated with samples from the B-14 trial [5]. Later, it was revealed that the assay also correlates with benefit from chemotherapy [6]. The assay is currently being evaluated in a randomized controlled trial to evaluate the benefit of chemotherapy *vs*. standard care in women with lymph node negative, ER-positive early stage BrCa with intermediate scores in the ongoing Trial Assigning IndividuaLized Options for Treatment (TAILORx) [7]. Overall, the Oncotype Dx test has good evidence from retrospective analyses of clinical trials and from small prospective observational studies, which have demonstrated that the test impacts treatment recommendations and the treatment received. Data from the clinical trial will further inform the optimal use of this assay.

MammaPrint^®^ is also a microarray-based test that measures the expression of 70 genes. The assay has been cleared by the US Food and Drug Administration (FDA) for the prognosis of patients with stage 1 or 2, node-negative, invasive BrCa where tumours are less than 5 cm in size [8]. It was developed and validated through several studies [9,10,11,12,13] and its clinical utility has been demonstrated in several retrospective studies [10,11] although uncertainty remains regarding the course of action when there is discordance between traditional clinicopathologic prognostic factor risk prediction and MammaPrint^®^. To address this, a prospective trial sponsored by the European Organization for Research and Treatment of Cancer is currently underway (Microarray in Node-Negative Disease May Avoid Chemotherapy Trial—MINDACT) [14].

Multi-gene assays such as Oncotype DX® and MammaPrint® have changed the understanding and management of BrCa, and represent a step forward in personalized medicine. The prognostic evidence from Oncotype DX® has led to the test being incorporated in clinical guidelines [13,14]. On the other hand, MammaPrint®, has achieved a regulatory approval by the FDA; however, given the nature of the supporting data, the evidence base is considered weaker [15]. In both cases the results of prospective trials are awaited. 

### 2.2. HER2/neu Expression and Response to Trastuzumab in BrCa

HER2/neu is a single prognostic marker that determines suitability for trastuzumab (Herceptin^®^) therapy in BrCa. Overexpression of the gene encoding for HER2/neu can occur in 25%–30% of BrCa [16], and is prognostic of poor outcomes. Having found trastuzumab as a drug that inhibits the overexpression of HER2/neu, a test to target use of the drug in the HER2-positive population would be predictive of improved outcomes.

The first test for HER2/neu over expression was developed as a companion diagnostic (CDx). An immunohistochemical (IHC) assay to select HER2-positive patients was implemented in trastuzumab’s phase III pivotal trial [16]. The clinical trial assay (CTA) was followed by the FDA-approved kit developed by DAKO (Glostrup, Denmark)—HercepTest^™^.

Concerns have been raised regarding HER2-testing’s performance relative to the CTA [17], and the different testing strategies depending on the methodology used [18]. The development of trastuzumab and HER2-testing, however, has been fundamentally important in the evolution of personalized medicine. Trastuzumab was approved first for use in metastatic BrCa, and subsequently for adjuvant treatment in early BrCa following a successful trial [19]. It was the most prominent early example of the successful translation of pharmacogenomics into clinical practice by integrating genomic technologies to tailor therapeutics to individual patients.

### 2.3. Use of EGFR Mutation Testing in Non-Small Cell Lung Cancer (NSCLC)

Lung cancer remains the most common cancer diagnosis in the world and mortality rates make it the leading cause of cancer-related death [20]. Treatment options in lung cancer depend upon the type of cancer, stage of disease, and patient health, and include surgery, radiation therapy, platinum based chemotherapy regimens, and epidermal growth factor receptor (EGFR) tyrosine kinase inhibitors (TKIs) [21].

Erlotinib (Tarceva^®^) and gefitinib (Iressa^®^) are two EGFR-TKIs used to treat patients with advanced NSCLC. Although gefitinib was first approved using phase II data, the subsequent phase III trial didn’t demonstrate survival benefit over best supportive care (BSC), thus, limiting the indication to patients who have previously benefited from gefitinib therapy [22]. Erlotinib was approved after a phase III trial (BR.21), which showed a survival benefit over BSC [23]. However, the benefit from erlotinib was only in a small subset of patients (approximately 10%–20%) leading researchers to hypothesize the potential for a molecular marker to be used to identify patients likely to benefit from EGFR-TKI treatment [24]. Subsequently, activating mutations in the EGFR gene were identified that have been shown to correlate with response and treatment benefit in the first line, maintenance, and 2^nd^/3^rd^ line settings. More recently, phase III clinical trials have provided evidence to support the use of EGFR mutational testing to select advanced NSCLC patients for first line treatment with EGFR-TKIs [25,26,27].

The identification of EGFR mutations and the association with outcomes for patients treated with gefitinib has in essence rescued gefitinib both clinically and commercially. Where once the prospects for gefitinib looked dire, it has now become a commonly used treatment in many markets. Though the prospects for erlotinib were never as bad as those for gefitinib, expanding its indication into first line, albeit in a small subset of NSCLC patients, could have substantial impact on its value to health systems and in global sales revenues. Additional drugs targeting tumors with *EGFR* mutations have been approved and more are in development [28]. 

### 2.4. Testing for KRAS Mutation in Colorectal Cancer (CRC)

The pathogenesis of CRC is closely related to the epidermal growth factor receptor (EGFR) pathway [29]. This pathway has been extensively studied allowing for the development of targeted therapies. Cetuximab (Erbitux^®^) and panitumumab (Vectibix^®^) are two monoclonal anti-EGFR antibodies. Although both drugs have demonstrated anti-tumor properties in CRC, response rates have been poor (~10%) [30,31]. Research has therefore been aimed at understanding and overcoming any mechanisms of resistance to increase this response rate. One area of investigation focuses on the discovery of any genetic aberrations downstream in the EGFR pathway that may be responsible for resistance to anti-EGFR antibodies. KRAS forms a vital part of the EGFR mediated pathway and KRAS mutations as a mechanism for the resistance of anti-EGFR antibodies have been established [32,33,34].

KRAS mutational testing in metastatic CRC is currently used to predict which patients will benefit from treatment with anti-EGFR monoclonal antibodies such as cetuximab and panitumumab. Through retrospective analyses of clinical trial data, the drug manufacturers established the clinical utility of KRAS mutational testing [35,36]. The original pivotal trial for cetuximab collected specimens to test for a different EGFR marker [37] and a strong association with KRAS was discovered in an *ex post* subgroup analysis [38]. The studies also demonstrated significant differences in progression-free survival (PFS) between wild-type KRAS patients and those with KRAS mutations. In a later study [39] benefit of Cetuximab reducing the risk of progression of mCRC was limited to KRAS wild-type tumours. The FDA expanded labeling and granted regulatory approval for cetuximab and panitumumab for the relevant subpopulations [40], albeit by accepting retrospective data to change the label for safety rather than efficacy reasons to avoid adverse events from treating non-responders.

KRAS mutational testing in metastatic colorectal cancer is routinely used to identify patients unlikely to benefit from treatment with anti-EGFR monoclonal antibodies. NCCN guidelines currently recommend evaluation of KRAS mutational status in CRC workups, and use of cetuximab and panitumumab is suggested for patients with wild-type KRAS tumors only. This example illustrates that given a convincing body of evidence even if generated *ex post* (not being a pre-specified primary analysis of trial data), both regulatory authorities and clinical guideline preparers are willing to consider such evidence as sufficient to impact regulatory approval and change recommended treatment protocols, respectively. 

### 2.5. BCR-ABL Monitoring Testing and the Use of Tyrosine Kinase Inhibitors (TKI)s in Chronic Myeloid Leukemia (CML)

CML is a cancer of the blood that is diagnosed through the detection of the “Philadelphia Chromosome” [41]. Effective treatment of CML with TKIs is essential to control the progression of the disease and improve patients’ overall survival. Imatinib (Gleevec^®^) has shown to be superior to interferon alfa plus low-dose cytarabine as first line therapy of CML [42]. Patients who don’t respond to treatment will progress to more severe disease phases thus having worse long-term prognosis [43,44]. In order to assess patient response to TKI treatment, regular monitoring and tyrosine kinase mutational analysis are recommended [41]. 

Monitoring of disease status and treatment response is based on the level of the fusion gene BCR-ABL in the patient’s peripheral blood. The clinically-significant level for suboptimal response (*i.e.*, major molecular response) in CML patients was established in the IRIS trial (International Randomized Study of Interferon and STI571) [45,46]. This has allowed for the development of BCR-ABL assays for the monitoring of disease predominately through laboratory-developed tests (LDTs) which in turn allowed for the routine implementation in clinical practice. Nevertheless, studies have shown accuracy and reliability shortcomings of laboratory-developed BCR-ABL tests. Such variability can have important implications for patient management and comparability of clinical research data, which lead to potentially undesirable health and economic impacts [47,48,49,50,51].

The clinical utility data from the IRIS trial has facilitated clinical decision-making and the development and adoption of BCR-ABL monitoring test. However, inter- and intra-laboratory variability may jeopardize this progress. Current efforts to address this issue, notably through the establishment of an International Scale anchored to values established and tested in the IRIS trial, or the development of commercial standardized test kits that have lower test variability and meet higher regulatory standards, have the potential to reduce the problem.

### 2.6. Clopidogrel and CYP2C19 Variants in Cardiovascular Disease

CYP2C19 is an enzyme involved in the metabolism of the oral antiplatelet agent clopidogrel (Plavix^®^). Clopidogrel and aspirin are standard of care to reduce the risk of major adverse cardiovascular events (MACE) in percutaneous coronary intervention (PCI). 

The importance of CYP2C19 genotyping in treatment using clopidogrel has been well documented. People who have inherited low CYP2C19 activity, which reduces the effectiveness of clopidogrel, will have an increase risk of MACE [52,53]. Therefore, in March 2010, the FDA introduced a “black box” warning for clopidogrel regarding the CYP2C19 activity; noting the availability of tests to determine CYP2C19 status, and of alternative antiplatelet medications [54]. For instance, the levels of active metabolite and clinical cardiovascular event rates in patients treated with prasugrel (Effient^®^) are not affected by common functional CYP variants [55]. Nonetheless, there has continued to be considerable controversy around the role of CYP2C19 activity. 

Although the predictive effect of CYP2C19 is clinically significant, testing is generally still not used clinically [56]. An observational study was begun to assess the practicalities of testing and the impact on patient outcomes [57]. Carrier status is usually determined by LDTs based on polymerase chain reaction (PCR), which is not feasible in a single, short consultation before prescribing clopidogrel to reduce the risk of a cardiovascular event. A new point-of-care (POC) genetic test, which can deliver results in about one hour, has been developed. The validated assay (SpartanRx^TM^) has been assessed in a prospective-randomized, proof-of-concept trial (RAPID GENE) with 200 patients [58]. The study provides clinical utility data on the appropriate treatment management of PCI patients in “real-time”, and facilitates the incorporation of genetic testing in clinical practice.

This is a potential case of how test manufacturers can fund the development of relevant clinical utility data, albeit with 200 patients. It remains to be seen, however, if clinicians and payers will react positively to these data and adopt testing in daily clinical practice.

### 2.7. Testing for the HLA-B*5701 Allele for HIV Treatment with Abacavir (ABC)

ABC was developed for use as part of a multi-drug regimen to treat people infected with HIV-1. ABC is highly effective and generally well tolerated; however, initial exploratory retrospective studies described a genetic association between HLA-B*5701 and ABC hypersensitivity (ABC-HSR) that affects between 2%–9% of patients treated [59].

These exploratory studies were motivated by two primary concerns: first, only a handful of patients developed ABC-HSR; and second, early epidemiologic analyses of the clinical trial data found racial differences in the risk of developing ABC-HSR. Based on retrospective analyses of trial data, the drug manufacturer identified the association between the HLA-B*5701 allele and ABC-HSR. The clinical utility of the prospective HLA-B*5701 screening on the incidence of ABC-HSR was assessed later in a RCT (PREDICT-1). The study found that prospective screening significantly reduces the overall frequency of ABC-HSR [59].

It is now well accepted that prospective screening for HLA-B*5701 can reduce the risk of ABC-HSR. In Europe and the US, HIV treatment guidelines recommend ABC be used only if patients have tested negative for HLA-B*5701. 

### 2.8. Testing for Viral Load Monitoring (VLM) and the Treatment of Hepatitis C

Standard treatment for patients infected with hepatitis C aims at clearing the virus with the combination of pegylated interferon and ribavirin. For some patients, however, sustained virological response (SVR) (*i.e.*, undetectable RNA at the end of follow-up, six months after completion of therapy) will not be achieved. Given the side effects and the high treatment costs, physicians want to identify virological non-responders as early as possible. As a result, VLM has become very important in treatment monitoring [60].

The clinical utility of VLM has been determined. For instance, a retrospective analysis on trial data [61] was conducted to determine if early virologic response (EVR) could be used to predict treatment response. It found that patients who failed to achieve an EVR after 12-weeks of treatment had no chance of having an SVR, even if they completed the additional nine-months of treatment. In addition, it concluded that the optimal definition of an EVR was a 2 log decrease in hepatitis C virus (HCV)-RNA levels after the first 12 weeks of treatment, regardless of HCV genotype. When assessing the economic impact of using EVR to identify and discontinue treatment of non-responders, the study reported cost savings in the population of patients infected with genotype 1 but not in those with genotypes 2 or 3. A different retrospective analysis supported the end of 12 weeks of treatment as a clinically relevant time point to decide the appropriateness for continuation of treatment [62]. It found that a viral load of above 30,000 IU/mL was 100% predictive of non-response in all patients. 

The clinical utility of the ability to predict treatment outcome based on VLM, and its economic impact, has been demonstrated. VLM using qualitative and quantitative assays is thus important for identifying subgroups of patients and an important tool in the treatment of hepatitis C.

### 2.9. PreDx^®^ Diabetes Risk Score (DRS) in Type-2 Diabetes

Type 2 diabetes has been associated with both genetic and environmental factors, and is believed to occur due to an interaction among these factors [63]. Nevertheless, diabetes can be prevented in many cases and the individuals at risk can be identified by utilizing common risk factors such as increased weight, familial history of diabetes, and high blood pressure [64]. Furthermore, recent studies have focused, on top of on clinical risk factors, on the added value of genotyping particular areas in the human genome that may identify those individuals at particular risk for diabetes [65].

The PreDx™ DRS is based on a blood assay and algorithm designed to examine genetic and protein biomarkers. It generates a numerical result between 1 (lowest risk) and 10 (highest risk), and estimates the patient risk for developing diabetes over the next five years. It is designed as an adjunctive test to complement but not replace existing clinical factor diagnoses and procedures, and is performed on a fasting blood sample [66]. The PreDX^®^ DRS was developed through a sub-cohort of the Inter99 cohort study—a large lifestyle intervention trial for cardiovascular disease in Denmark [67]. Through its (retrospective) validation the PreDX^®^ DRS was found to be significantly better than most current methods of determining risk including overnight fasting glucose test [68].

While the PreDX^®^ DRS has been shown to be comparable to the gold standard (an oral glucose tolerance test), it remains to be validated in a large population and gain widespread acceptance. The Inter99 study included people from Denmark only; these results may not be representative of all populations and, thus, validation work in different populations may be required. In addition, a key issue will be provider willingness to use this test in addition to fasting glucose and payer willingness to provide reimbursement for this test.

## 3. Benefits from MDx to Patients and the Health Care System

Each of these nine case studies demonstrate that a companion MDx can:

- provide information to patients and health care providers;
- allow for a targeting of treatments or other interventions to a subset of the population despite differences in whether they are prognostic, predictive, or used for monitoring;
- offer the potential for the health system to deliver more health gain to patients.

In one of these case studies—HLA-B*5701 allele testing for the use of ABC for HIV—use of an MDx avoids adverse reactions in patients and thus reduces other health care costs on the health care system, by avoiding the costs associated with treating them. In the case of OncotypeDX^®^ and MammaPrint^®^ the treatment regimen can be refined to avoid unnecessary chemotherapy saving cost and improving patient quality of life [69]. In three cases, drug costs are reduced by targeting the population with higher probability to respond to a specific treatment (HER2 testing for trastuzumab treatment in women with HER2-positive BrCa, EGFR mutation testing in NSCLC patients for EGFR-TKI treatment-decisions, and testing for KRAS mutation in CRC to target the use of cetuximab and panitumumab). Of course, MDx tests have an associated direct cost, and drug companies may increase prices for targeted treatments, and the evidence supporting their benefits and the net impact after taking account of all costs is not always as strong or unambiguous as payers, clinicians, patients, and indeed drug and diagnostic manufacturers would like. However, there is good evidence for several of these case studies of the potential for patient stratification to deliver health gains while being cost-effective [70,71,72,73]. 

## 4. Practical Issues and Obstacles to the Use of MDx in Health Care Systems: The Strength of the Evidence Base

The nine cases in this paper illustrate the potential for the effective use of MDx tests to deliver improvements in patient health and in the efficiency of health care delivery. They show the potential for well-designed studies to allow for the demonstration of clinical utility (and the subsequent development of economic evidence) such that the value of targeting therapy can be demonstrated to payers. However, the strength of the evidence has an important impact on acceptance and uptake of an MDx test. Table 1 sets out the sources of evidence for our nine case studies. 

Table 1 shows that the predominant funders of evidence on the clinical utility of MDx are drug developers (as part of a co-development) and public research bodies. Diagnostic companies play a limited role. Payers were funding one study, but it was terminated when ownership changed. Whilst the limited role of payers is expected given the public good nature of evidence, the low level of investment by diagnostic manufacturers seems to be surprising. 

## 5. Conclusions

The nine case studies address significant health problems with varying impact. In order to support health care decision-making, and stimulate innovation and new technology uptake, evidence of clinical utility needs to be present, albeit not necessarily via an RCT. Our cases suggest that decision-makers are willing to consider studies other than RCTs (e.g., retrospective cohort studies) if well designed. Eight of our nine case studies involve using tests to make decisions about the use of drugs. PreDx^®^ DRS is about prevention more generally.

**Table 1:** Sources of clinical utility evidence for decision-making in the nine case studies.

Marker	Main Study Design	Study size (patient numbers)	Sponsor	Decision-making Impact
Breast cancer recurrence (a) Oncotype DX^®^ and (b) MammaPrint^®^ (Prognostic/predictive in BrCa)	Retrospective RCT cohorts RCTs	(a) 688 [4] +651 [5] +895 [6] (b) Prognostic: 117 [9] +295 [10] +307 [11] +123 [12], Predictive: 241 [13] (a) 11248 [7] (b) 6600 [14]	Diagnostic manufacturer Public research body Public research bodies	Generating clinical utility can yield inclusion in clinical guidelines and positive reimbursement decisions at a favourable price for test developers.
HER2 (Trastuzumab in metastatic and early stage BrCa)	RCTs	469 [16] 3676 [19]	Drug developer Drug developer	Positive reimbursement decision for drug-diagnostic in a specific subpopulation based on health gains and cost-effectiveness.
EGFR mutations (1st line TKI treatment in NSCLC)	RCTs RCTs	1217 [25] 165 [26] 173 [27]	Drug developer Drug developer	Drug rescued because of a predictive, *ex post* companion diagnostic. Obtained first line indication
KRAS mutations (Anti-EGFR monoclonal antibodies in CRC)	Retrospective cohort analysis of an RCT	1198 [39]	Drug developer and Public research body	Decision-makers are willing to consider evidence generated *ex post* as sufficient to change recommended treatment protocols.
BCR-ABL transcript (TKI treatment in CML)	RCT	1106 [42]	Drug developer	Actionable, clinical information allowed for full incorporation into clinical guidelines; however, issues with inter- and intra-laboratory variability may impact patient management thus health outcomes.
CYP2C19 (Clopidogrel in ACS)	Retrospective RCT cohort+Healthy volunteers Prospective cohort study Proof-of concept RCT	1477+162 [52] 4471 [57] (Terminated early)187 [58]	Public research body Payer Diagnostic manufacturer	The clinically significant and validated predictive effect would allow for health care efficiencies in the treatment of ACS. New POC test could improve implementation in clinical practice.
HLA-B*5701 (ABC in HIV)	Retrospective case control RCT	408 [59] 1956 [59]	Drug developer Drug developer	Prospective screening for first-line treatment is cost-effective in specific sub-populations. Treatment guidelines recommend abacavir only if patients have tested negative for HLA-B*5701.
Viral load (Pegylated interferon and ribavirin in hepatitis C)	Retrospective analyses of RCT data	1016 [61] 260 [62]	Drug developer Public research body	Testing becomes fundamental for predicting treatment outcome, reducing treatment side-effects and avoiding futile treatment and subsequent costs in non-responding patients.
PreDx® DRS (Risk in Type 2 diabetes)	Retrospective analysis of a sub-cohort of a lifestyle interventional trial	6784 [67,68]	Assay developer (trial funded by a public research body)	Although the score has been proven significantly better than other available methods and similar to the gold standard, uptake has been very limited. Data may not be generalisable to the whole population, and payers may not want to cover the test in addition to fasting glucose testing.

The development of the clinical utility evidence is feasible through either or both (i) drug developer or diagnostic manufacturer sponsored studies and (ii) research sponsored by payers and public bodies. Our case studies suggest it is less clear that the diagnostic industry can undertake the work necessary to provide evidence as to the clinical utility and economic value of its products. Oncotype DX^®^ and MammaPrint^®^ may be the best examples of diagnostic manufacturers engaged in the development of clinical utility and economic data to support use of their tests. Even here, further prospective generation of RCT data is being undertaken by public research bodies. In the case of CYP2C19 testing, a diagnostic manufacturer has funded a small proof-of-concept trial. However, it remains to be seen what impact this has on payers. There are examples of diagnostic manufacturers commissioning small observational studies (for example, in the case of a test for coronary artery disease) [74]. Even small clinical studies are, however, the exception rather than the rule. In general, it appears that test reimbursement levels may not be sufficient to encourage diagnostic manufacturers to invest in evidence collection via large studies (RCT or observational). Tests are often assigned codes and/or prices that are not value-related. In addition, the regulatory and data protection frameworks for diagnostics do not encourage the development of evidence of clinical value. For example, large studies involving diagnostics would generate information that can be used by competing test providers who have not invested in evidence collection. Lack of good evidence reduces payer willingness to reimburse tests and clinician willingness both to use them at all, and, when they are used and paid for, to act on the results. As a consequence, opportunities to generate value from personalized medicine (in terms of cost savings or health gains) may be lost. Thus, whilst drug developer funding of co-development is important, and payers and public bodies should fund research of value on MDx, creating better incentives for diagnostic companies to bring clinical utility evidence to the market is a key public policy issue.
